# The Public Health Implications of China’s Travel Ban for Smoking on Trains

**DOI:** 10.18502/ijph.v49i10.4709

**Published:** 2020-10

**Authors:** Minxing ZHAO

**Affiliations:** Department of Intellectual Property Law, Baoding University, Hebei, China

## Dear Editor-in-Chief

As of May 1^st^, 2018, China’s travel banned for smoking on trains took effect. The new regulation stipulates that any individual who smokes on China’s high-speed trains or in the smoke-free area of regular trains could be barred from traveling for 180 days (1). Since train is the most popular vehicle of passenger transport in a country with 1.4 billion people, the 180-day travel ban is believed to be the toughest penalty for smoking in public places. The newest penalty for smoking on trains has several important public health implications.

The travel ban can have real effects on health and well-being of non-smoking passengers. This travel ban is especially meaningful to control smoking on regular trains because smoking has already been completely banned on all high-speed trains. There are designated smoking areas on regular trains and smoking is prohibited in smoke-free areas. But non-compliance often happens. Although smoking in smoke-free area will face a fine of up to 2,000 Chinese Yuan (300 US Dollars), the monetary penalty cannot effectively deter smoking. Non-smoking passengers are frequently exposed to second-hand smoke, which has led to the first smoking ban litigation in 2017. If the travel ban is strictly enforced, smoking passengers will control their smoking behaviour and non-smoking passengers will be less exposed to second-hand smoke, which is known to cause cancer, other diseases and death (2). Admittedly, the travel-ban alone cannot guarantee clean air on all regular trains, but it is a recognized method to deter smoking on trains.

While the effects of travel ban on public health remain to be seen, the rationale underpinning the travel ban shows that policies and legislations that have positive impact on public health may not be driven by public health considerations. Smoking on trains is not a public issue in China until the fast development of high-speed trains in the past decade. Smoking ban on high-speed trains is not primarily intended to reduce the harm of second-hand smoking but for the safety of train transportation because smoking will cause high-speed trains to make emergency stops (3). This indicates that concerns over the safety of high-speed trains are far more justifiable than concerns over public health in banning smoking on high-speed trains. Due to increasing violations of smoking ban, the railway authority introduced travel ban. The logic of smoking ban also applies to travel ban. The travel ban is also not out of public health considerations and smoking on trains is punished as an untrustworthy social mis-deed under China’s new “social credit” system (4). Whereas such measures are not in the name of promoting public health, they are in fact more acceptable to the public because they transcend the need to balance the interests of smokers and non-smokers.

The travel ban is a significant move to ease the way for national tobacco control policy. As the world’s largest tobacco producer and consumer, China’s tobacco control measures have lagged far behind the harm that tobacco has caused to public health. Since China ratified the WHO Framework Convention for Tobacco Control in 2005, many tobacco control measures have been adopted. Despite their limited effectiveness, none of them has reached the magnitude of having national public health implications. For a long time, the lack of a national smoking-free law is often criticized by tobacco control advocates. The travel ban implemented by China’s state-own railway company could make the tobacco control measure a national one given the high volume of passengers travelling by train every year around the country.

In conclusion, the travel ban makes smoke-free regulations more powerful. However, the public health implications of the travel ban are determined by how effectively the regulation is implemented. In addition to legal requirements, it is recognized that there are variations in law enforcement in many parts of China, which also have an important bearing on the potential public health benefits. While it is helpful for tobacco control on trains, the travel ban may not increase the public health awareness of smokers and non-smokers because the rationale underpinning the new penalty is public safety and personal social credit, not public health. It may further the conflicts between smokers and government when travellers are banned from travelling by trains.

## References

[1] China to bar people with bad ‘social credit’ from planes, trains. Reuters [newspaper on the Internet]. 2018 Mar 16 [cited 2018 May 2]. Available from: *https://www.reuters.com/article/us-china-credit/china-to-bar-people-with-bad-social-credit-from-planes-trains-idUSKCN1GS10S*

[2] Asomaning K Miller DP Liu G Wain JC Lynch TJ Su L Christiani DC (2008). Second hand smoke, age of exposure and lung cancer risk. Lung Cancer, 61( 1): 13–20. 18191495 10.1016/j.lungcan.2007.11.013 PMC2515267

[3] Ma C. Harsher punishment for smoking on high-speed trains. China Daily [newspaper on the Internet]. 2016 Aug 16 [cited 2018 May 3]. *http://www.chinadaily.com.cn/china/2016-08/16/content_26490229.htm*

[4] Laband J. How can individuals, companies be limited by bad social credit in China? China Business Review [Internet]. 2017.

